# Solid-Phase Synthesis of Head to Side-Chain Tyr-Cyclodepsipeptides Through a Cyclative Cleavage From Fmoc-MeDbz/MeNbz-resins

**DOI:** 10.3389/fchem.2020.00298

**Published:** 2020-04-22

**Authors:** Gerardo A. Acosta, Laura Murray, Miriam Royo, Beatriz G. de la Torre, Fernando Albericio

**Affiliations:** ^1^CIBER-BBN, Networking Centre on Bioengineering, Biomaterials and Nanomedicine, University of Barcelona (UB), Barcelona, Spain; ^2^Department of Organic Chemistry, University of Barcelona, Barcelona, Spain; ^3^Institute of Advanced Chemistry of Catalonia (IQAC-CSIC), Spanish National Research Council (CSIC), Barcelona, Spain; ^4^Associated Unit, Spanish National Research Council-University of Barcelona (CSIC-UB), Barcelona, Spain; ^5^KwaZulu-Natal Research Innovation and Sequencing Platform (KRISP), School of Laboratory Medicine and Medical Sciences, College of Health Sciences, University of KwaZulu-Natal, Durban, South Africa; ^6^Peptide Science Laboratory, School of Chemistry and Physics, University of KwaZulu-Natal, Durban, South Africa

**Keywords:** cyclothiodepsipeptides, heterodetic cyclic peptides, homodetic cyclic peptides, native chemical ligation, solid-phase peptide synthesis

## Abstract

Cyclic depsipeptides constitute a fascinating class of natural products. Most of them are characterized by an ester formed between the β-hydroxy function of Ser/Thr -and related amino acids- and the carboxylic group of the *C*-terminal amino acid. Less frequent are those where the thiol of Cys is involved rendering a thioester (cyclo thiodepsipeptides) and even less common are the cyclo depsipeptides with a phenyl ester coming from the side-chain of Tyr. Herein, the preparation of the later through a cyclative cleavage using the Fmoc-MeDbz/MeNbz-resin is described. This resin has previously reported for the synthesis of cyclo thiodepsipeptides and homodetic peptides. The use of that resin for the preparation of all these peptides is also summarized.

## Introduction

Peptides are an important class of Active Pharmaceutical Ingredients (APIs) (Henninot et al., 2018; Lau and Dunn, 2018; Al Shaer et al., 2020; de la Torre and Albericio, 2020). They show excellent chemical and biological diversity, as well as high activity and specificity, low toxicity, low accumulation in tissues, and minimization of drug-drug interactions. Furthermore, the production of peptides ready for use in humans is relatively affordable thanks to the development of two complementary technologies, namely solid-phase peptide synthesis (SPPS) and reversed-phase high-performance liquid chromatography (RP-HPLC). The former was first described by Bruce Merrifield (Merrifield, 1963), and later refined by several groups, while the latter, founded on the pioneering work of Jim Waters (McDonald, 2006), allows the purification of complex synthetic crude peptides. Both technologies can be used to prepare peptides to fulfill research and industrial requirements (Zompra et al., 2009; Rasmussen, 2018).

**Figure 1:**
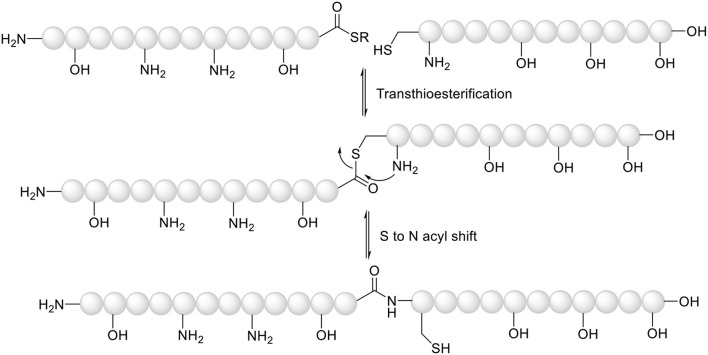
Mechanism of native chemical ligation.

Peptide synthesis can be briefly defined as the proper combination of protecting groups (Isidro-Llobet et al., 2009) and coupling reactions—the latter based exclusively on the activation of the carboxylic group (El-Faham and Albericio, 2011). Although there are a large number of coupling reagents that cleanly render the active species (El-Faham and Albericio, 2011), the development of the native chemical ligation (NCL) technique by Stephen Kent brought about a breakthrough in the field (Dawson et al., 1994). NCL involves the preparation of an unprotected *C-*thioester peptide that allows reaction with an unprotected *N-*terminal Cys to render first the thioester involving the thiol of the Cys, which rearranges to form the natural amide bond (Figure 1). This technique has allowed the synthesis of difficult, large peptides, and proteins (Camarero and Muir, 1999; Camarero et al., 2001; Miller et al., 2003; Avital-Shmilovici et al., 2013; Okamoto et al., 2014a,b; Boll et al., 2015; Pala-Pujadas et al., 2018; Snella et al., 2018; Spengler et al., 2018; Ashhurst et al., 2019; Sun and Brik, 2019; Jbara et al., 2020; Serra et al., 2020).

**Figure 2:**
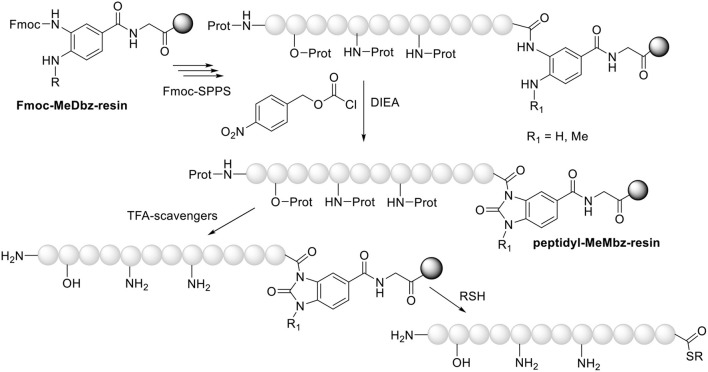
Preparation of peptide thioester via Fmoc-MeDbz/MeNbz-resins.

One of the keys to the success of the NCL approach is the preparation of unprotected *C-*thioester peptides. Although these compounds were first synthesized using the *tert-*butyloxycarbonyl (Boc)/benzyl (Bzl) strategy, several methods have been proposed for the same purpose using the friendlier fluorenylmethoxycarbonyl (Fmoc)/*tert-*butyl (tBu) approach. In this case, the main problem to overcome is the lack of stability of the thioester to the piperidine used in each step to remove the Fmoc group. In this regard, the thioester should be formed after elongation of the peptide. Although several linkers have been developed for this purpose (Camarero et al., 2004; Mulder et al., 2014; Kawakami et al., 2016; Morisaki et al., 2016; Olivier et al., 2017; Rao and Liu, 2017; Shelton et al., 2017; Flood et al., 2018), the 3-(Fmoc-amino)-4-(methylamino) benzoic acid (Fmoc-MeDbz, the acronym derives from *N-*me*thyl-*d*iamino*b*enzoic* a*cid*) and/or its non-methylated analog developed by Blanco-Canosa and Dawson are optimal (Blanco-Canosa and Dawson, 2008; Blanco-Canosa et al., 2015) Fmoc-MeDbz is anchored to an amino resin. After removal of the Fmoc group, peptide elongation proceeds smoothly using normal coupling reagents (the electronic deactivation of the aromatic ring by the growing peptide chain and the presence of the Me in the second amino group impede the development of a second peptide chain on the *N-*Me-amino). Once the peptide has been elongated, the resin is reacted with *p*-nitrophenyl chloroformate, followed by cyclization in basic media [*N,N-*diisopropylethylamine (DIEA)] to render *N*-acyl-*N'*-methyl-benzimidazolinone (MeNbz)-resin. As an activated *N*-acylurea (*N*-acyl-*N'*-methylurea), this resin is susceptible to being a leaving group when under the attack of a nucleophile such as a thiol and therefore to rendering, in this case, the peptide thioester (Figure 2). This resin is considered a “safety catch,” in the terms described by Patek and Lebl, namely stable throughout peptide elongation and then activated to be labile to a given reagent (Patek and Lebl, 1999).

This MeNbz-resin has also been used by Stockdill's group to synthesize *C-*terminal-modified peptides (Arbour et al., 2017, 2018a,b). In this case, the linker is released through an intermolecular reaction by the action of nucleophiles such as alcohols, thiols, amines, and even amino acids.

**Figure 3:**
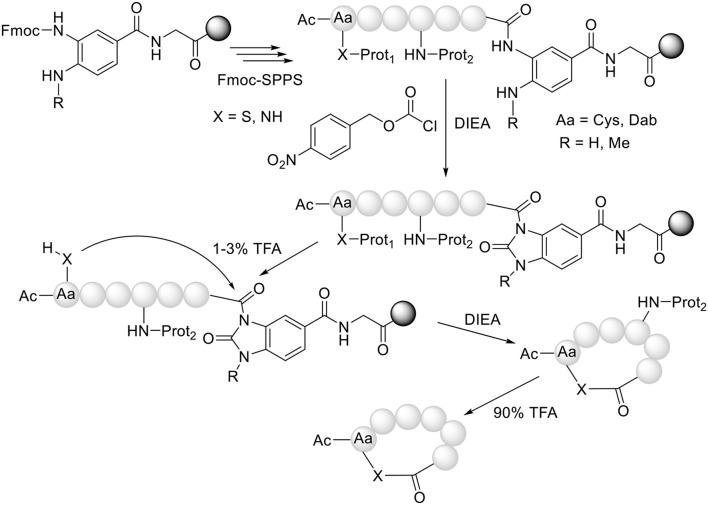
Synthesis of head to side-chain cyclothiodepsipeptides (X = S) and cyclohomodeticpeptides (X = NH).

Our group (Acosta et al., 2017) and Olsen's (Gless et al., 2017; Gless and Olsen, 2018) have used the same strategy but in an intramolecular mode for the preparation of cyclothiodepsipeptides. In this strategy, after the formation of the MeNbz-activated species, the protecting group of the side-chain of a Cys residue is removed and the free thiol attacks the peptidyl-MeNbz-resin to render the cyclic thioester, with concomitant release of the peptide from the resin (Figure 3). A further advantage of this “cyclative cleavage” approach is that only the cyclic peptide is released from the resin. In this regard, those lineal sequences that have not converted to the cyclic structure remain unaltered on the resin, thereby simplifying the crude product and thus the purification step.

Our group has extended this “cyclative cleavage” to the preparation of homodetic cyclic peptides, where the nucleophile that causes the cleavage is an amino function, preferably from the side-chain of a peptide containing a basic amino acid (Abdel Monaim et al., 2018). In this case, the formation of the “head to side-chain” cyclic peptide is more favorable than the corresponding “head to tail” cyclic peptide, presumably due to the higher basicity of the ω-amino vs. the α-amino function (Figure 3).

Stockdill's group has recently reported an elegant strategy combining the two previous methods for the preparation of a “head to tail” cyclic peptide containing a Cys residue (Arbour et al., 2019). Thus, the formation of the cyclic thiodepsipeptide takes place by the attack of the thiol of a *N-*terminal Cys on the peptidyl-MeNbz-resin, after which the cyclic thiodepsipeptide rearranges to form the homodetic head to tail cyclic peptide.

An important class of cyclo heterodetic peptides are the head to side-chain cyclodepsipeptides, where a residue of Ser, Thr, or other β-hydroxy amino acid forms an ester bond with the carboxylic group of *C-*terminal amino acid (Pelay-Gimeno et al., 2013). In our hands, the application of cyclative cleavage on *N-*acyl-*N'-*MeDbz-resin for the preparation of Ser cyclodepsipeptides has proved unsuccessful, even when using strong bases such as NaH (results not shown).

Finally, less common but very intriguing peptides of the same family are those containing a phenyl ester coming from the phenol of a Tyr. These Tyr-cyclodepsipeptides are found in the fengycin family, which are lipodepsipeptides isolated from Bacillus strains (Sieber and Marahiel, 2003). Their syntheses are a challenge due to the inherent lability of the phenyl ester bond, which should be formed during the last steps of the synthetic strategy. Feliu and Planas' group developed an elegant strategy (Rosés et al., 2016; Roses et al., 2018), based on the following: incorporation of the first amino acid through the side-chain with the carboxylic acid protected in the form of the allyl ester; elongation of the peptide chain; formation of the phenyl ester with an *N-*Alloc-amino acid; treatment with Pd(0) to remove the allyl and the Alloc protecting groups; on-resin cyclization; and global deprotection and cleavage from the resin.

Here we report on a further application of the Fmoc-MeDbz/MeNbz-resin strategy for the synthesis of Tyr-cyclodepsipeptides.

## Results and Discussion

**Figure 4:**
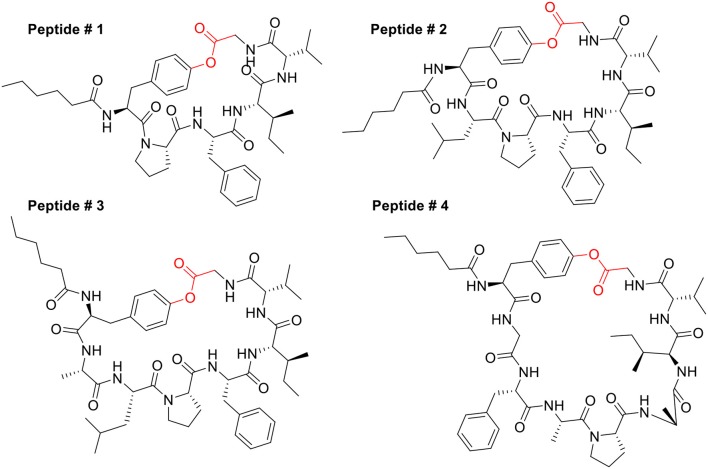
Structures of model peptides # 1–4.

As the aromatic ring of the Tyr side chain should confer more rigidity to the pending unit than the side chains of Cys or Lys/Orn/Dab/Dap studied in previous works (Acosta et al., 2017; Abdel Monaim et al., 2018), we first investigated the relevance of cyclodepsipeptide size with respect to facilitating the cyclization. Thus, model peptides with 6, 7, 8, and 9 amino acids were prepared, which correspond to cycles of 23, 26, 29, and 32-member rings, respectively (Figure 4). The sequence of these peptides was based on our previous works, related to the preparation of cyclo thiodepsipeptides and homodetic peptides as well (Acosta et al., 2017; Abdel Monaim et al., 2018). The peptide was built on a Fmoc-MeDbz-Gly-Rink-ChemMatrix resin with Fmoc/tBu amino acids using *N,N'*-diisopropylcarbodiimide (DIC) and OxymaPure in *N,N*-dimethylformamide (DMF) as coupling method (Subiros-Funosas et al., 2009). Tyr protected as Fmoc (α-amino) and 2-chlorotrityl (ClTrt) (phenol side-chain) was incorporated as the last amino acid. The Fmoc was removed with piperidine-DMF (2:8) and the amino function was acylated with hexanoic acid (Hx-OH). The Hx-Tyr(CTrt)-peptidyl-MeDbz-Gly-Rink-ChemMatrix resin was activated with *p*-nitrophenyl chloroformate in anhydrous DCM, followed by cyclization with 0.5 M DIEA in DMF for 45 min to render Hx-Tyr-peptidyl-MeNbz-resin with concomitant appearance of an intense yellow color due to the release of the *p-*nitrophenol. The peptide resin was then treated with a low concentration solution of trifluoroacetic acid (TFA) in DCM to remove the ClTrt protecting group of the phenol of Tyr. The peptide-resin was then washed thoroughly to ensure that no acid remained. The final cyclative cleavage occurred using 10% DIEA in DCM. The solutions, including the washings with DCM, were collected and concentrated. Cold diethyl ether (DEE) was added to facilitate the precipitation, and then centrifuged and decanted. The remaining solid was dissolved in CH_3_CN-H_2_O (1:1) and lyophilized.

**Figure 5:**
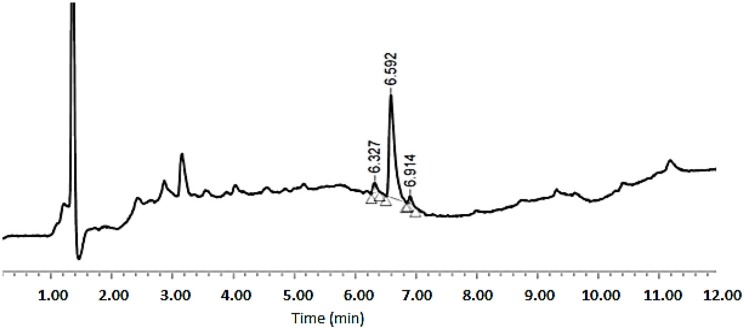
HPLC analysis of crude peptide # 2 on a XBridge BEH130 C_18_ 3.5 mm, 4.6 × 100 mm column. Eluents, A: H_2_O with 0.045% of TFA; B: ACN with 0.036% of TFA. Gradient: 5–100% B into A in 8 min, 1.0 mL/min, 220 nm 25°C.

**Figure 6:**
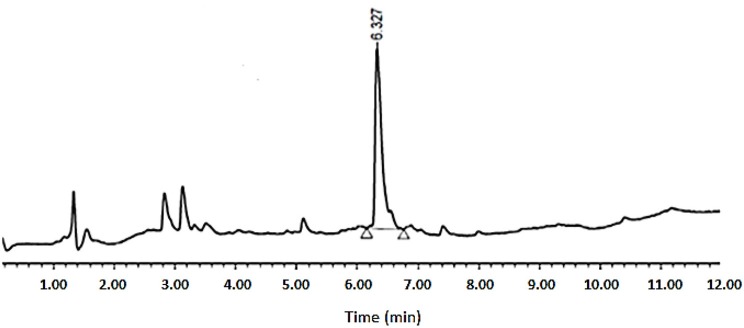
HPLC analysis of crude peptide # 3 on XSelect C_18_ 3.5 mm, 4.6 × 50 mm. Eluents, A: H_2_O with 0.1% of formic acid; B: ACN with 0.07% of formic acid. Gradient: 5–100% B into A in 8 min, 1.6 mL/min, 220 nm, 50°C.

As shown in Table 1, peptides 1 and 4, the smallest and the largest cycles, were not observed. In these cases, cyclization was attempted at 60°C with 10% DIEA in DMF, but no cyclic peptides were detected. However, peptides # 2 and # 3, containing 7 and 8 amino acids, respectively, showed a major peak in HPLC (Figures 5, 6) and the mass was corroborated by HPLC-MS (Supplementary Figures 3, 6).

**Table 1:** Summary of the results obtained with the model peptides.

**Peptide #**	**No. of AA**	**Peptidyl-resin**	**Cleaved Peptide Expected/Found mass**	**Cyclization**
1	6	Hx-**Y**PFIVG-MeNbz-resin	774.4/-	No
2	7	Hx-**Y**LPFIVG-MeNbz-resin	888.1/888.4	Yes
3	8	Hx-**Y**ALPFIVG-MeNbz-resin	959.2/959.6	Yes
4	9	Hx-**Y**GFAPAIVG-MeNbz-resin	974.2/-	No

*Hx, Hexanoyl*.

It is difficult to generalize about peptide cyclization; however, peptides containing the same number of amino acids as peptide # 1, having Cys and Dab instead of Tyr, were able to cyclize (Acosta et al., 2017; Abdel Monaim et al., 2018). It is therefore possible to confirm that the phenol of the Tyr side chain imposes more restrictions than Cys or Dab residues. These limitations can be translated in either an inability to achieve the cyclative cleavage or/and the instability of the Tyr-cyclodepsipeptide once formed. As the linear peptides were not clearly detected, the most plausible explanation is the first one.

**Figure 7:**
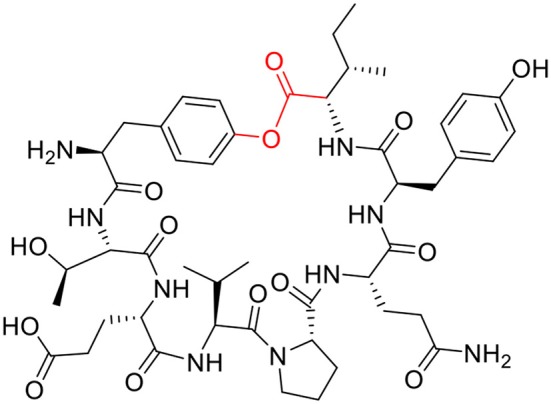
Structure of BPC822 peptide.

Next, the synthesis of the antifungal peptide BPC822 (Figure 7), first synthesized by Feliu and Planas' group, was attempted using the current methodology based on the use of the Fmoc-MeDbz/MeNbz-resin.

BPC822 contains eight amino acids similar to peptide # 3, and the phenyl ester is formed between the carboxylic group of the Ile and the phenol of the side-chain of Tyr. The involvement of the Ile, a β-substituted reside could add further difficulty to the formation of the ester. Thus, the Boc-Tyr(ClTrt)-Thr(*t*Bu)-Glu(O*t*Bu)-Val-Pro-Gln(Trt)-D-Tyr(*t*Bu)-Ile-MeDbz-Gly-ChemMatrix-resin was constructed on a resin of reduced loading (0.33 mmol/g) to facilitate the cyclization. The resin was activated by treatment with *p*-nitrophenyl chloroformate in anhydrous DCM, followed by 0.5 M DIEA in DMF for 1 h with concomitant development of an intense yellow color due to the release of the *p-*nitrophenol to render the peptidyl-MeNbz-resin. The ClTrt, protecting group of the side-chain of the Tyr, was removed by short treatments with diluted solutions of TFA in DCM, as above for the model peptides.

**Figure 8:**
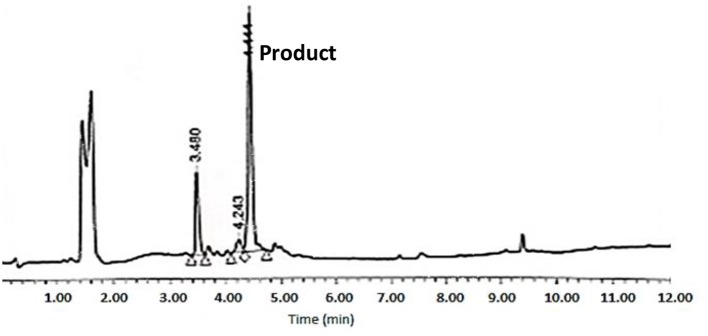
HPLC analysis of crude BPC822 peptide cyclized at 60°C on a XBridge BEH130 C_18_ 3.5 mm, 4.6 × 100 mm column. Eluents, A: H_2_O with 0.045% of TFA; B: ACN with 0.036% of TFA. Gradient: 5–100% B into A in 8 min, 1.0 mL/min, 220 nm 25°C. The peak at 3.48 min could not be identified by MS.

**Figure 9:**
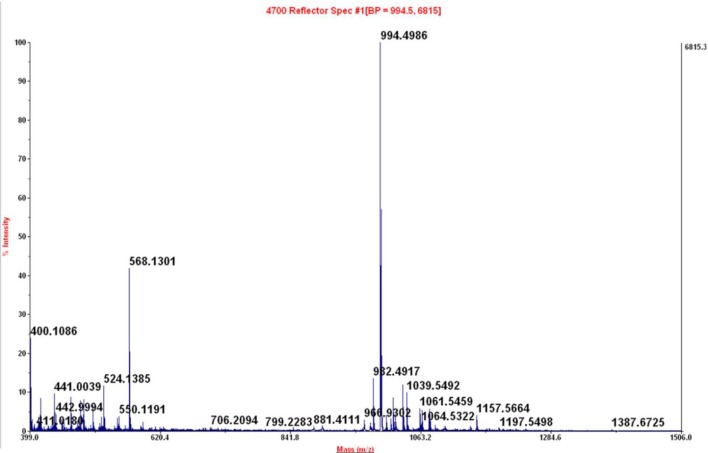
Maldi-TOF of peak at 4.44 min of crude reaction peptide BPC822 obtained by heating at 60°C for 30 min. Calculated mass 993.48; mass found: [M + H]^+^ = 994.4986.

The cyclative cleavage was first attempted with 10% DIEA at room temperature, but after the corresponding work-up (see below) no product was obtained. The same reaction was then repeated at 60°C for 30 min and under microwave-assisted heating (100 w) at 80°C for 15 min. In both cases, the DMF was evaporated and the remaining oil was treated with concentrated solutions of TFA-H_2_O-TIS (95:2.5:2.5) for 1 h to remove the remaining side-chain protecting groups. After partial evaporation of the TFA, the final product was then precipitated with cold DEE, centrifuged, decanted, and lyophilized. Although the two methods rendered the target peptide, the HPLC corresponding to the cyclative cleavage at 60°C (Figure 8) showed a much cleaner profile than that carried out under microwave conditions (Supplementary Figure 12). The final peptide was characterized by LC-MS and Maldi-TOF (Figure 9 and Supplementary Figures 9, 10, 11 and Table 1). In some cases, during the lyophilization and/or the posterior analysis, hydrolysis of the cyclic peptide was detected, thereby reinforcing the idea of the partial instability of head to side-chain Tyr-cyclodepsipeptides.

## Conclusions

Here, the “safety catch” Fmoc-MeDbz/MeNbz-resin developed by Blanco-Canosa and Dawson for the preparation of linear peptide thioesters was investigated to synthesize “head to side-chain” cyclodepsipeptides through a cyclative cleavage. While this strategy proved unsuccessful for the preparation of those peptides involving Ser in the formation of the ester bond, even using strong bases, it allowed the preparation of Tyr-cyclodepsipeptides using DIEA as a base. In the case of unhindered *C-*terminal amino acids, the cyclative cleavage took place at room temperature. When the *C-*terminal amino acid was more hindered, the reaction occurred at 60°C. This technology represents a friendly method for the preparation of these fascinating peptides.

## Experimental

See Supplementary Information for a more detailed description of the full process. Herein, the key steps are described.

### Fmoc-MeDbz-Gly-ChemMatrix-resin and SPPS

Although any amino resin can be used, aminomethyl ChemMatrix resin increases the success of the synthesis of these complex peptides (Garcia-Martin et al., 2006). To ensure quantitative incorporation of the Fmoc-MeDbz linker, a spacer of Gly is introduced to minimize steric hindrance caused by the polymeric matrix. The incorporation of the building blocks, linker and protected amino acids took place smoothly using 3 equiv. of each component, namely carboxylic acid, DIC, and OxymaPure in DMF. The incorporation first protected acid on the H-MeDbz-resin was carried out with HATU-DIEA due to the poor nucleophilicty of the benzyl amine present in the linker. Fmoc was removed with piperidine-DMF (2 × 5 min). Washings with DMF and DCM were intercalated during the treatments.

### Formation of the Activated *N*-acyl-*N'*-MeNbz-resin

Acylation of the NMe aniline moiety was achieved by treatment with 4-nitrophenyl chloroformate (5 equiv.) in anhydrous DCM for 1 h. Two treatments were carried out to achieve quantitative yield. Although an acyl chloride was used, the reaction was done in the absence of base. After resin filtration and washing, 0.5 M of DIEA in DMF was added for 45 min to render the cyclic urea. The release of 4-nitrophenol is shown by a strong yellow color, which indicates the formation of the cyclic urea.

### Cyclative Cleavage

After the formation of the methylurea, the ClTrt of the phenol of Tyr was removed with TFA-triisopropylsilane (TIS)-DCM (2.5:2.5:95, 5 × 2 min washes). The resin was then washed thoroughly with DCM, DMF, and DCM, to ensure that no acid remained. The final cyclisation step occurred using 10% DIEA in DCM or DMF either at room temperature, at 60°C, or under microwave conditions, and the resin was further washed with DCM. The solution of 10% DIEA, including the DCM washings, was collected and evaporated to dryness. In the case of BCP822 peptide, the residue was treated for 1 h with TFA-TIS-DCM (95-2.5-2.5) to remove the remaining protecting groups and then evaporated to dryness.

In both cases, cold DEE was added in order to facilitate the precipitation of the peptide, which was then centrifuged and decanted. The remaining solid was dissolved in CH_3_CN-H_2_O (1:1) and lyophilized.

## Data Availability Statement

The datasets generated for this study are available on request to the corresponding author.

## Author Contributions

Experimental part was performed mainly by GA and LM under the supervision of the rest of co-authors. The manuscript was written with contributions from of all the authors. All authors have given approval to the final version of the manuscript.

## Conflict of Interest

The authors declare that the research was conducted in the absence of any commercial or financial relationships that could be construed as a potential conflict of interest.
