# Supplementary material for: Solid-Phase Synthesis of Head to Side-Chain Tyr-Cyclodepsipeptides Through a Cyclative Cleavage From Fmoc-MeDbz/MeNbz-resins
Source: Front Chem. 2020 Apr 22;8:298. doi: 10.3389/fchem.2020.00298 (PMC7189019; doi:10.3389/fchem.2020.00298)
Supplement: Supplementary file 1 [file Table_1.DOCX]

**SUPPLEMENTARY INFORMATION**

**Solid-Phase Synthesis of** **Side-Chain to Tail Tyr-Cyclodepsipeptides through a Cyclative Cleavage from Fmoc-MeDbz/MeNbz resins**

Gerardo A. Acosta,^1,2,3,4^ Laura Murray,^1,2^ Miriam Royo,^2,3,4^ Beatriz G. de la Torre,^5,6^ Fernando Albericio^1,2,3,4,6,*^

^1^CIBER-BBN, Networking Centre on Bioengineering, Biomaterials and Nanomedicine, Marti i Franques 1-11, University of Barcelona (UB), 08028 Barcelona, Spain

^2^Department of Organic Chemistry, Marti i Franques 1-11, University of Barcelona, 08028 Barcelona, Spain

^3^Institute of Advanced Chemistry of Catalonia (IQAC-CSIC), Spanish National Research Council (CSIC), Jordi Girona 18-26, 08034 Barcelona, Spain

^4^Associated Unit CSIC-UB, Jordi Girona 18-26, 08034 Barcelona, Spain

^5^KwaZulu-Natal Research Innovation and Sequencing Platform (KRISP), School of Laboratory Medicine and Medical Sciences, College of Health Sciences, University of KwaZulu-Natal, Durban 4041, South Africa

^6^Peptide Science Laboratory, School of Chemistry and Physics, University of KwaZulu-Natal, Durban 4001, South Africa

**Figure 1.**  Structure of Peptide # 2


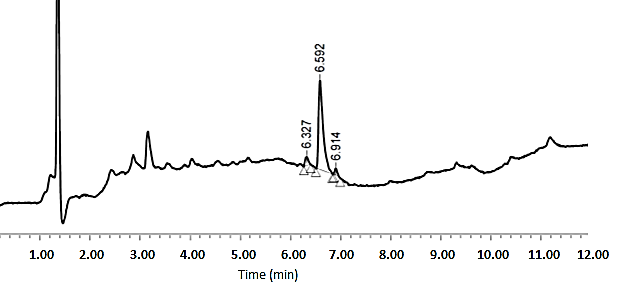


**Figure 2.** HPLC analysis of crude peptide # 2. Peak at 6.592 min corresponds to the target peptide

**Analysis Conditions: Column:** XBridge BEH130 C_18_ 3.5 mm, 4.6 x 100 mm. **Eluents:** A: H_2_O with 0.045% of TFA B: ACN with 0.036% of TFA**. Gradient:** 5-100 % B into A in 8 minute. **Flow:** 1.0 mL/min**. Temperature:** 25 ºC**. Detection Wavelength**: 220 nm


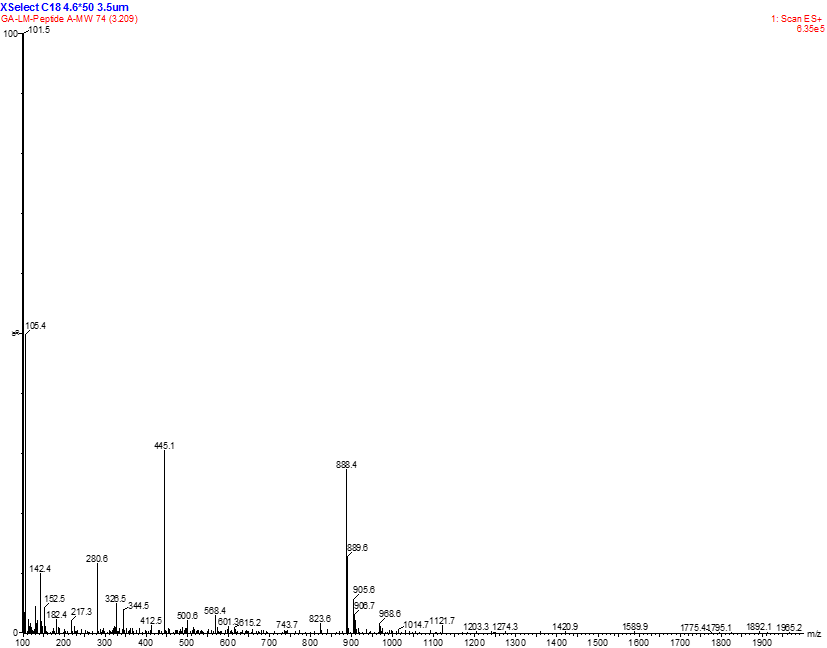


**Figure 3.**  Mass spectrum crude peptide # 2. Calculated mass: 888,1. Found massa: [M+H]^+1^ = 888,4; [M+NH_4_]^+1^ = 905,6.

**Analysis Conditions:** Column: XSelect C_18_ 3.5 mm, 4.6 x 50 mm. Eluents: A: H_2_O with 0.1% of formic acid B: ACN with 0.07% of formic acid. Gradient: 5-100 % B into A in 8 minute. Flow: 1.6 mL/min. Temperature: 50 ºC.

**Figure 4.**  Structure of Peptide # 3


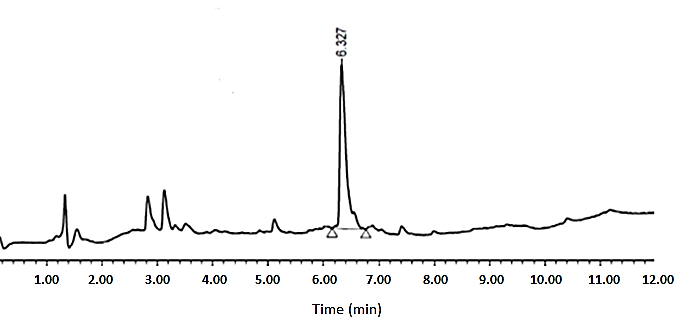


**Figure 5.** HPLC analysis of crude peptide # 3. Peak at 6.327 min corresponds to the target peptide.

**Analysis Conditions: Column:** XBridge BEH130 C18 3,5 mm, 4,6 x 100 mm. **Eluents:** A: H_2_O with 0,045% of TFA B: ACN with 0,036% of TFA**. Gradient:** 5-100 % B into A in 8 minute. **Flow:** 1,0 mL/min**. Temperature:** 25 ºC**. Detection Wavelength**: 220 nm


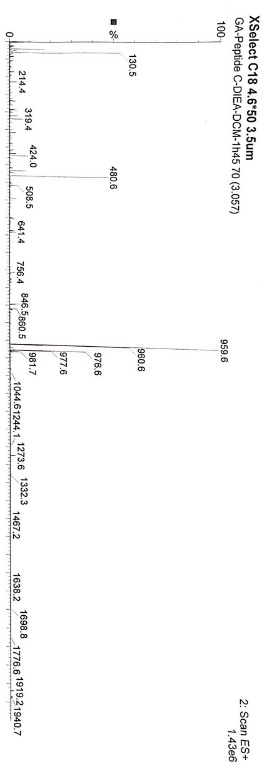


**Figure 6.** HPLC-MS analysis of crude peptide # 3. Calculated mass: 959,2. Found massa: [M+H]^+1^ = 959,6; [M+2H]^+2^ = 480,6 and [M+NH_4_]^+^ =976,6.

**Analysis Conditions: Column:** XSelect C_18_ 3.5 mm, 4.6 x 50 mm. **Eluents:** A: H_2_O with 0.1% of formic acid B: ACN with 0.07% of Formic acid**. Gradient:** 5-100 % B into A in 8 minute. **Flow:** 1.6 mL/min**. Temperature:** 50 ºC**. Detection Wavelength**: 220 nm.

**Figure 7.**  Structure of Peptide BPC822

**Solid-Phase Synthesis of BPC822 (Fmoc/*t*Bu strategy)**

***Materials and Methods***

Protected amino acids were supplied by Iris Biotech (Marktredwitz, Germany). ChemMatrix resin and OxymaPure were gifts from PCAS BioMatrix (Quebec, Canada) and Luxembourg Biotech (Ness Ziona, Israel), resectively. Solvents for peptide synthesis and RP-HPLC equipment were obtained from Scharlau (Barcelona, Spain). TFA was supplied by KaliChemie (Bad Wimpfen, Germany). Other chemicals were obtained from Aldrich (Milwaukee, WI) and were of the highest commercially available purity. All commercial reagents and solvents were used as received.

RP-HPLC characterization was performed using Waters Alliance 2695 (Milford, MA) chromatography systems, equiped with Waters 995 photodiode array detector and ESI-MS Waters Micromass ZQ. Semipreparative RP-HPLC was performed on a Waters (Milford, MA) chromatography system using Symmetry C18 (3 × 10 cm, 5 μm) columns.

Solid-phase synthesis was carried in polypropylene syringe fitted with a polyethylene disk. Washings (4 x 0.5 min after each coupling and Fmoc removal) and piperidine-DMF (2:8) (2 x 5 min) treatment are carried with 1 mL of the corresponding solvent per gram of resin.

***Synthetic procedures***

H-Rink Amide ChemMatrix resin (0.3 g, 0.33 mmol/g) was washed with DMF, DCM, and DMF. Fmoc-Gly-OH (3 equiv., 0.3 mmol) is coupled with DIC (3 equiv., 0.3 mmol) and OxymaPure (3 equiv., 0.3 mmol) in DMF (0.5 mL) at room temperature for 1 hr.

*Glycine is used as spacer to avoid the steric hindrance between the resin and the Fmoc-MeDbz linker and facilitating synthetic process.*

The Fmoc group was removed and the resin washed with DMF and DCM. The 3-(Fmoc-amino)-4-(methylamino) benzoic acid (Fmoc-MeDbz) (3 equiv., 0.3 mmol) was coupled with DIC/OxymaPure (3 equiv., 0.3 mmol each) in a DMF-*N*-methylpyrrolidone NMP (1:1) (1 mL) for 2 hr, then the Kaiser test gave negative. The Fmoc group was removed and Fmoc-Ile-OH (3 equiv., 0.3 mmol) was incorporated using HATU (3equiv., 0.3mmol) and DIEA (5 equiv., 0.5 mmol) in DMF (0.5 mL) for 1 hr at room temperature.

*This coupling requires strong conditions due to the low nucleophilicity of the benzyl amine present in the linker.*

The rest of amino acids were coupled using DIC-OxymaPure (3 equiv., 0.3 mmol) in DMF (0.5 mL) for 45 min at room temperature. In all cases the Kaiser test was negative. The Fmoc of the last Tyr was removed and the α-amino function was reprotected with di-*tert*-butyldicarbonate (Boc_2_O) (3 equiv., 0.3 mmol) in DMF (0.5 mL) for 45 minutes at room temperature.

*The N^α^-amine of Tyr is better to be reprotected in form of Boc. The presence of the free amine would will interfere with the cyclic depsipeptide formation, because it self could act as nucleophile rendering the cyclic head-to-tail peptide.*

*Activation of the linker*

4-Nitrophenyl chloroformate (5 equiv., 0.5 mmol) in anhydrous DCM (0.5 mL) was added to the resin and stirred for 1 hr at room temperature and the treatment was repeated once. The resin was washed with DMF, DCM and Isopropanol, DMF and DCM.

*Acylurea Formation*

0.5 M DIEA in DMF (1.5 mL) was added to the resin and stirred at room temperature for 1 hr.

*The appearance of an intense yellow color shows the formation of acylurea due to the release of 4-nitrophenol.*

*Deprotection of the Tyr side chain*

The resin was washed with a mixture of TFA-TIS-DCM (2.5:2.5:95, 5 x 2 min), followed by washing the resin with DCM, DMF, and DCM, to remove all traces of acid.

*Cyclization and global deprotection*

The peptide resin (80 mg) was treated with 10% DIEA in DMF (1 mL) for 1 hr at room temperature. Then, an aliquot of the solution was analyzed by HPLC and no peptide was observed. Then, the treatment with 10% DIEA in DMF was repeatd at 60 °C for 30 min. The HPLC analysis of an aliquot of the solution showed the appearance of a peak corresponding to the target peptide. The solution was filtered off and the resin was washed with DMF and DCM. The collected solution was evaporated to dryness, DCM was added, and the solution was evaporated again to dryness (3 times). The residue was treated with TFA-TIS-DCM (95-2.5-2.5) (1 mL) for 1 hr to remove the protecting groups. Then, the TFA solution was evaporated to dryness, cold DEE was added, and the precipitated was dissolved in ACN-H_2_O (1:1) and lyophilized rendering 10.2 mg of crude product (yield 42.5 %).

Alternatively, other aliquot of the peptide resin (80 mg) was treated with 10% DIEA in DMF (1 mL) and left to react under microwave-assisted heating (100 watts of potencia) at 80 ºC of temperature for 15 min. The HPLC analysis of an aliquot of the solution showed several peaks. Then, the solution was filtered off and the same work-up than earlier was followed to render 9.5 mg of crude product (yield 38.3%).


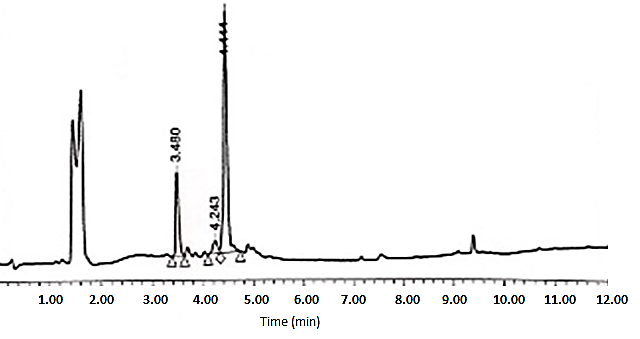


**Figure 8.**  HPLC analysis of crude BPC822 peptide, at 60ºC. Peak at 4.444 min corresponds to the target peptide. The peak at 3.480 min could not be identified by MS.

**Analysis Conditions: Column:** XBridge BEH130 C_18_ 3.5 mm, 4.6 x 100 mm. **Eluents:** A: H_2_O with 0.045% of TFA B: ACN with 0.036% of TFA**. Gradient:** 5-100 % B into A in 8 minute. **Flow:** 1.0 mL/min**. Temperature:** 25 ºC**. Detection Wavelength**: 220 nm

**Figure 9.**  HPLC-MS crude reaction, peak rt = 4.456, peptide BPC822 obtained by heating at 60 ° C for 30 min. Calculated mass 993.48; mass found: [M + H]^+^ = 994.6; [M + 2H]^+2^ = 498.1

**Analysis Conditions: Column:** XSelect C_18_ 3.5 μm, 4.6 x 50 mm. **Eluents:** A: H_2_O with 0.1% of formic acid B: ACN with 0.07% of Formic acid**. Gradient:** 5-100 % B into A in 8 minute. **Flow:** 1.6 mL/min**. Temperature:** 50 ºC**.**


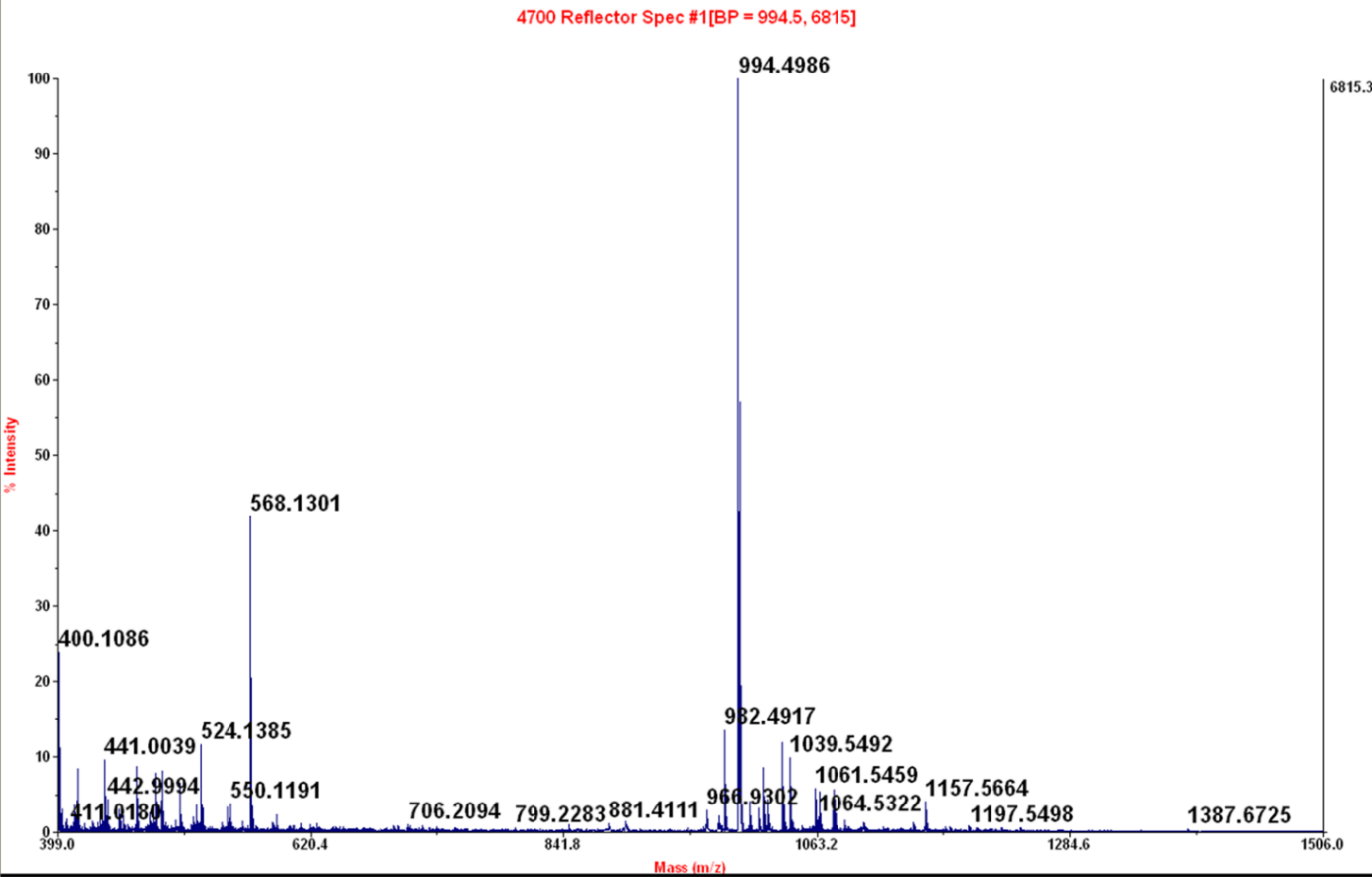


**Figure 10.**  Maldi-TOF of peak at 4.44 min of crude reaction peptide BPC822 obtained by heating at 60°C for 30 min. Calculated mass 993.48; mass found: [M + H]^+^ = 994.4986.

**Figure 11.**  Maldi-TOF MS-MS crude reaction, peak 2, peptide BPC822 obtained by heating at 60 ° C. The 8 fragment ions corresponding to the amino acids present in the molecule are shown. Ionization Source: MALDI (Matrix Assisted Laser Desorption Ionization) Solid State Laser (Nd:YAG) (355nm, 200Hz, 3-7ns pulse). Analyzer: TOF/TOF (Time of Flight) in reflector mode or linear mode depending on the m/z. Matrix CHCA (α-cyano-4-hydroxy cinnamic acid) (10mg/mL H_2_O-CH_3_CN 1:1 0.1%TFA).

| Ions(Charge) | Pro | Gln | Tyr | Ile | Tyr | Thr | Glu | Val |
| --- | --- | --- | --- | --- | --- | --- | --- | --- |
| a ions (+1) | **70.066** | **198.124** | **361.188** | **474.272** | **637.335** | **738.383** | **867.425** | **966.494** |
| b ions (+1) | 98.061 | **226.119** | **389.183** | **502.267** | **665.330** | **766.378** | **895.420** | 994.489 |
| b-H2O (+1) | -- | **--** | **--** | **--** | -- | 748.367 | 877.410 | 976.478 |
| b+H2O (+1) | -- | **--** | **--** | **--** | -- | -- | -- | -- |
| C ions (+1) | 115.087 | **243.146** | **406.209** | **519.293** | 682.356 | 783.404 | 912.447 | -- |
| X ions (+1) | -- | **941.426** | **813.367** | **650.304** | 537.220 | 374.156 | 273.109 | 144.066 |
| Y ions (+1) | 1012.499 | **915.446** | **787.388** | **624.324** | 511.240 | 348.177 | 247.129 | 118.087 |

**Table** 1. Theoretical ions and ions found, the latter are marked in bold. Confirming the sequence of the peptide

**Figure 12.**  HPLC analysis of crude BPC822 peptide, at 80ºC using microwave, Rt of the product 4.456. Peaks at Rt = 3.493 and 4.221 could not be identified by MS.

**Analysis Conditions: Column:** XBridge BEH130 C_18_ 3.5 mm, 4.6 x 100 mm. **Eluents:** A: H_2_O with 0.045% of TFA; B: ACN with 0.036% of TFA**. Gradient:** 5-100 % B into A in 8 minute. **Flow:** 1.0 mL/min**. Temperature:** 25 ºC**. Detection Wavelength**: 220 nm
